# Uptake and effectiveness of a mobile application for real-time reporting and quality assurance of decentralized SARS-CoV-2 testing in Uganda

**DOI:** 10.3389/fpubh.2023.1053544

**Published:** 2023-06-01

**Authors:** Hellen Nansumba, Proscovia Nambuya, Jackson Wafula, Namutebi Laiton, Rigveda Kadam, Olukunle Akinwusi, Khairunisa Suleiman, Paula Akugizibwe, Isaac Ssewanyana

**Affiliations:** ^1^Central Public Health Laboratories, Ministry of Health, Kampala, Uganda; ^2^Foundation for Innovative New Diagnostics (FIND), Geneva, Switzerland

**Keywords:** COVID-19, decentralized diagnosis, digital, data, surveillance, quality assurance

## Abstract

**Background:**

Effective management of the COVID-19 pandemic required rapid expansion of diagnosis. The introduction of antigen tests presented an opportunity to decentralize testing, but raised challenges with ensuring accurate and timely reporting of testing data, which is essential to guide the response. Digital solutions can help address this challenge and provide more efficient means of monitoring and quality assurance.

**Methods:**

Uganda’s existing laboratory investigation form was digitized in the form of an Android-based application, eLIF, which was developed by the Central Public Health Laboratory and implemented in 11 high-volume facilities between December 2021 and May 2022. The app enabled healthcare workers to report testing data via mobile phone or tablet. Uptake of the tool was monitored through a dashboard that enabled real-time visibility into data being transmitted from sites, as well as qualitative insights from site visits and online questionnaires.

**Results and discussion:**

A total of 15,351 tests were conducted at the 11 health facilities during the study period. Of these, 65% were reported through eLIF, while 12% were reported through preexisting Excel-based tools. However, 23% of tests were only captured in paper registers and not transmitted to the national database, illustrating the need for increased uptake of digital tools to ensure real-time data reporting. While data captured through eLIF were transmitted to the national database within 0–3 days (min, max), data transmitted through Excel were transmitted in within 0–37 days (min, max), and data for paper-based reporting took up to 3 months. The majority of healthcare workers interviewed in an endpoint questionnaire responded that eLIF improved timeliness of patient management, and reduced reporting time. However, some functions of the app were not successfully implemented, such as providing random selections of samples for external quality assurance and enabling seamless linkage of these data. Challenges arose from broader operational complexities, such as staff workload, frequent task-shifting and unexpected changes to facility workflows, which limited adherence to the envisioned study procedures. Ongoing improvements are needed to adjust to these realities, to strengthen the technology and support to healthcare workers using it, to optimize the impact of this digital intervention.

## Introduction

Since the first reported case of SARS-CoV-2 in Uganda in March 2022, over 169,000 infections and 3,620 deaths have been registered, as of August 2022 (1, 2). The pandemic has manifested through multiple waves of infections, each associated with the emergence of variant strains that may increase transmission rates (3, 4). To ensure timely and agile responses to this rapidly evolving virus, expansion of diagnostic coverage—along with rapid access to reliable data on testing outcomes—is essential (5).

Polymerase chain reaction (PCR) targeting various SARS-CoV-2 conserved gene regions is the gold standard for diagnosis, due to its high sensitivity and specificity (6). However, the method is expensive, has a long turnaround time to results, and requires specialized laboratory facilities and personnel skills (7). In the first year of the pandemic response, this caused delays in detection and missed opportunities for timely interventions (7). The introduction of rapid antigen diagnostic tests (Ag-RDTs)—which are less costly and have fewer requirements in terms of infrastructure, biosafety and skill—presented exciting opportunities to increase access to testing and strengthen surveillance, particularly at the peripheral level of health systems in low-and middle-income countries (LMICs) (8).

In September 2020, following field evaluation, Uganda adopted SARS-CoV-2 Ag-RDTs for use in health facilities and community testing. However, uptake has been relatively low, accounting for 6% of the 3.98 million tests COVID-19 tests conducted in Uganda by December 2022 (9). Data management has been one of the challenges associated with expansion of decentralized rapid diagnostic testing and implementation of electronic health interventions is constrained by the complexity of Uganda’s health system design (10).

Timely reporting of SARS-CoV-2 infections is a critical pillar of the pandemic response (5). Daily reporting is more feasible with centralized PCR testing in laboratories that typically have elaborate laboratory information management systems. It is difficult to realize timely and accurate reporting on Ag-RDT testing at numerous lower-level health facilities that lack electronic information systems. In settings where paper-based data management is used, long lags in the transmission of Ag-RDT testing data have been noted. For example, in South Africa, it was estimated that the median time between testing and reporting for Ag-RDTs was 29.7 days in the public sector (11).

In LMIC settings, digital solutions have provided a means to ensure that data could be reliably collected from decentralized testing sites (12, 13). While digital solutions have often served a similar function in other disease responses in Uganda in the past (12, 13), the national e-Health and data management strategy highlights that these tools have often been fragmented, with limited scale-up outside of individual projects (14).

Mobile networks cover almost all of Uganda, including rural and remote areas since 2012 and 60.53 per 100 people in the country were mobile phone subscribers by 2020 (14, 15). As such, digital tools have considerable potential to transform the timeliness and efficiency with which health data is reported and used to inform decisions. For optimal scalability of such tools especially in remote areas, they would need to be accessible via mobile phone, so as to minimize infrastructure requirements, and integrated with national health data systems for seamless transfer of patient information.

In the context of the COVID-19 response, there was also an opportunity to use digital tools to support external quality assurance of Ag-RDT testing, as at the time of their introduction, the field performance of these tests was not routinely monitored in Uganda. Establishment of end-to-end patient records that linked outcomes of both rapid and confirmatory PCR tests could enable such post-market surveillance to be routinely conducted. Inclusion of symptom data in these patient records also offered the possibility of deeper insights into the relationship between clinical factors and SARS-CoV-2 outcomes.

In the first year of the pandemic response, COVID-19 testing data were largely captured by facilities in a paper-or Excel-based Laboratory Information Form (LIF), from which data was subsequently uploaded to the national results dispatch system (RDS). This required multiple steps of capturing, transcribing and transmitting data—and for Excel-based reporting, access to a computer in the facility. This study aimed to evaluate the uptake and effectiveness of a digitized form accessed through an Android-based app, eLIF (electronic LIF), that could be used by healthcare workers on mobile devices and integrated with RDS to enable real-time reporting and monitoring of testing.

Sustainable entry of digital tools into public health systems requires identifying and working with enablers and constraints of successful adoption, which vary between and within countries. A recent review of lessons from the implementation of digital solutions for community and primary healthcare workers in different African countries highlighted wide variation in the effectiveness of these interventions, with the most common issues being infrastructure (connectivity and uninterrupted power supply) and digital literacy in the health workforce (16). However, with these and other considerations being highly context-specific, there is a need to design localized evaluations of the adoption of digital technologies, in order to understand key success factors as well as opportunities for improvement.

## Methods

### Study design

This was a cross-sectional multi-site study conducted in 11 health facilities, selected on the basis of having high COVID-19 testing volumes with limited access to PCR testing and long result turnaround time.

The study included all suspected SARS-CoV-2 cases presenting to participating facilities who were eligible for COVID-19 testing according to the national testing guidelines. Patients received the standard of care with the study interventions focusing on data capture and selection for external quality assurance (EQA).

A mixed-methods analysis was used to evaluate feasibility, uptake and acceptability of the app. This comprised analysis of quantitative data on utilization, site visits for direct observation of implementation, and structured questionnaires administered to healthcare workers.

### Overview of digital system

eLIF presented a sustainable option for digitizing rapid testing data as it was fully developed and hosted by Uganda’s Central Public Health Laboratory (CPHL), allowing for easier customization and alignment with broader digital systems than if the technology was owned by a third party.

**Figure 1:**
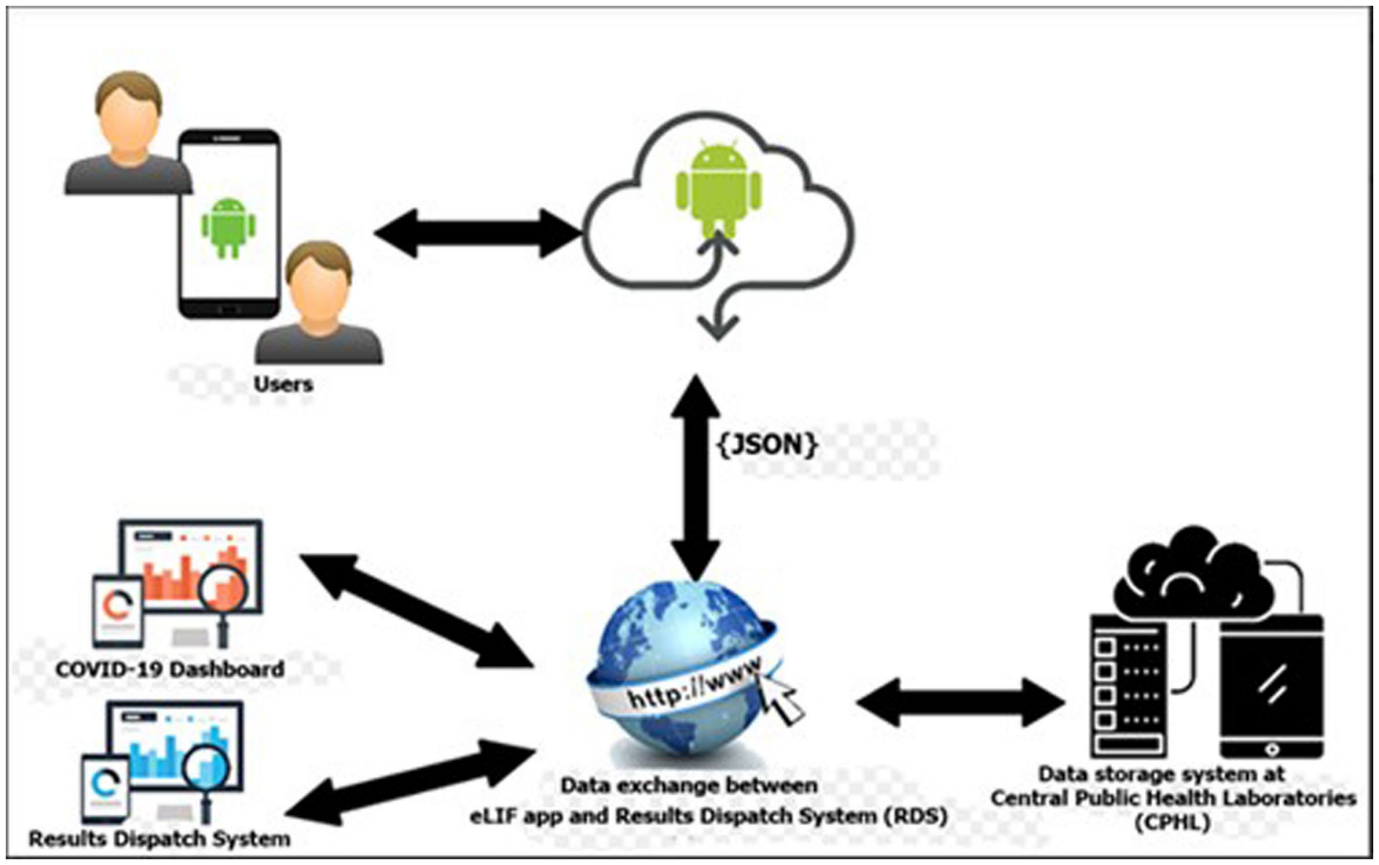
eLIF architecture. CPHL, Central Public Health Laboratory; eLIF, electronic Laboratory Information Form; JSON, JavaScript Object Notation; RDS, national results dispatch system.

The main components of the eLIF system are the users, access devices, mobile application, database server and web services. Information such as user’s details and patient information are stored in the databases. Web services are used to transfer information between the mobile application and the web applications (RDS and COVID-19 Dashboard). The mobile application provides different interfaces for users based on the activities they need to perform. Figure 1 describes the high-level architecture of the mobile application for eLIF system.

The eLIF mobile application uses JavaScript Object Notation (JSON) format for data transmission from the web server to the mobile application for log-in. JSON is used because it is supported by most major programming languages and is used commonly as a preferred information exchange format between web clients and servers (Figure 1).

### Development process

The development of eLIF was led by Uganda’s CPHL ICT team, with technical inputs from Foundation for Innovative New Diagnostics (FIND), the global alliance for diagnostics. The development was done in line with principles of digital development (17). The seven phases of the software development lifecycle were as follows: planning, definition of user requirements (Laboratory Investigation Form), design and prototyping, software development, testing, deployment, operations and maintenance. The study focused on the last two phases of this lifecycle.

### Mobile app design

Once a health worker logged in, eLIF presented the “*Enter investigation data*” tab to capture patient information. This included patient identifiers (only accessible to facility or CPHL personnel, to protect confidentiality), demographic and clinical information, and sample type.

Once this was completed and a sample taken for the rapid test, the patient’s information was available in a “*pending results*” tab. Healthcare workers could capture investigation data for additional patients while waiting for the test to complete (approximately 15 min), then return to the “pending results” tab to enter the test result and date. eLIF was also designed to randomly select and flag every tenth negative sample for referral to EQA, which included confirmatory PCR testing as well as genomic sequencing to determine which variant of SARS-CoV-2 caused the infection. All positive samples were also referred for EQA. The overall target for EQA (4000) was determined based on feasibility. These estimates also informed the frequency of random selection of negative Ag-RDTs (1 in 10) to ensure sufficient volume was reached. After the tester assigned a result, the app would flag a request for EQA by PCR for selected samples, with a provision to scan the barcode attached to the Ag-RDT sample for ease of linking records between the rapid testing site and the PCR lab.

Once results had been entered, an “*Edit Recently Entered Results*” allowed healthcare workers to correct any errors within 5 min of the result entry. Thereafter, it was not possible to make changes to the test result, which is a standard measure used by CPHL to ensure data integrity. It was assumed any errors would be detected within this time frame, after which the client may have departed and the test cleared away. The application also included a verification quality control step, displaying the test result and identifiers before the submission of the final result. A “*view submitted results*” tab enabled healthcare workers to search any records that had been captured in eLIF using patient identifiers. eLIF was linked to RDS for automated transmission of all data into the national COVID-19 data repository. Through the “*Go to Results Dispatch System*” tab, healthcare workers could print official test reports for patients where required.

In addition to capturing patient data, eLIF also included functions to support implementation monitoring. Through the “*Enter Logistics Data*” tab, healthcare workers could report on stock status of the supplies used for testing, such as Ag-RDTs and swabs. The “*COVID-19 Dashboard*” tab also gave them access to the study dashboard, which was used to monitor disease trends and performance indicators.

### COVID-19 dashboard design

The study dashboard, developed as an open-source web application, was hosted within the CPHL server and allocated a public IP address to enable access to offsite users. The dashboard is responsive and accessible across mobile devices with various display dimensions. Facility users and approved study personnel with log-in credentials could access a study-specific section of the national dashboard,^1^ where more detailed data and analytics from the participating facilities were hosted. This study data could be viewed via download of an Excel dataset with raw, disaggregated and de-identified records for all patients whose data had been entered into eLIF.

The dashboard also included performance monitoring tabs that provided aggregated epidemiological and operational indicators from the facilities, with automated alerts to flag if any indicators went outside a pre-defined “acceptable” range and thus prompt further investigation by CPHL. A log of queries submitted by study facilities, with notes on the status and how these had been addressed, were also visible on the dashboard.

### User requirements

eLIF was available in the Google Play Store (“eLIF-UGANDA”) for use by healthcare workers in participating study facilities. To access eLIF, healthcare workers needed a mobile device that used an Android operating system and had available memory of 110 MB. Once installed, they were required to log in using the individual username and password assigned by the CPHL team to protect access to data and ensure that data could be linked to a specific health worker and facility. While internet connectivity was required for transmission of data to RDS, data capture and other app functions did not require an active connection, to enable use at health facilities with poor internet connection.

### Privacy and confidentiality

To ensure confidentiality of information, each user was given restricted access and sharing of accounts was discouraged. A second level of authentication within eLIF was highly recommended. During login, a code was sent to the mobile device via text message, which was mandatory to input in order to access the system.

### Training and roll-out

A training of trainers was conducted at CPHL, following which trainers were dispatched to facilities to train laboratory and clinical healthcare workers from participating facilities on the use of the app and the study workflow. Each facility was provided with two mobile devices and internet bundles to support data capture, with healthcare workers also encouraged to install and use the app on their personal devices where needed, and provided with internet data bundles to facilitate this.

### Monitoring implementation

**Figure 2:**
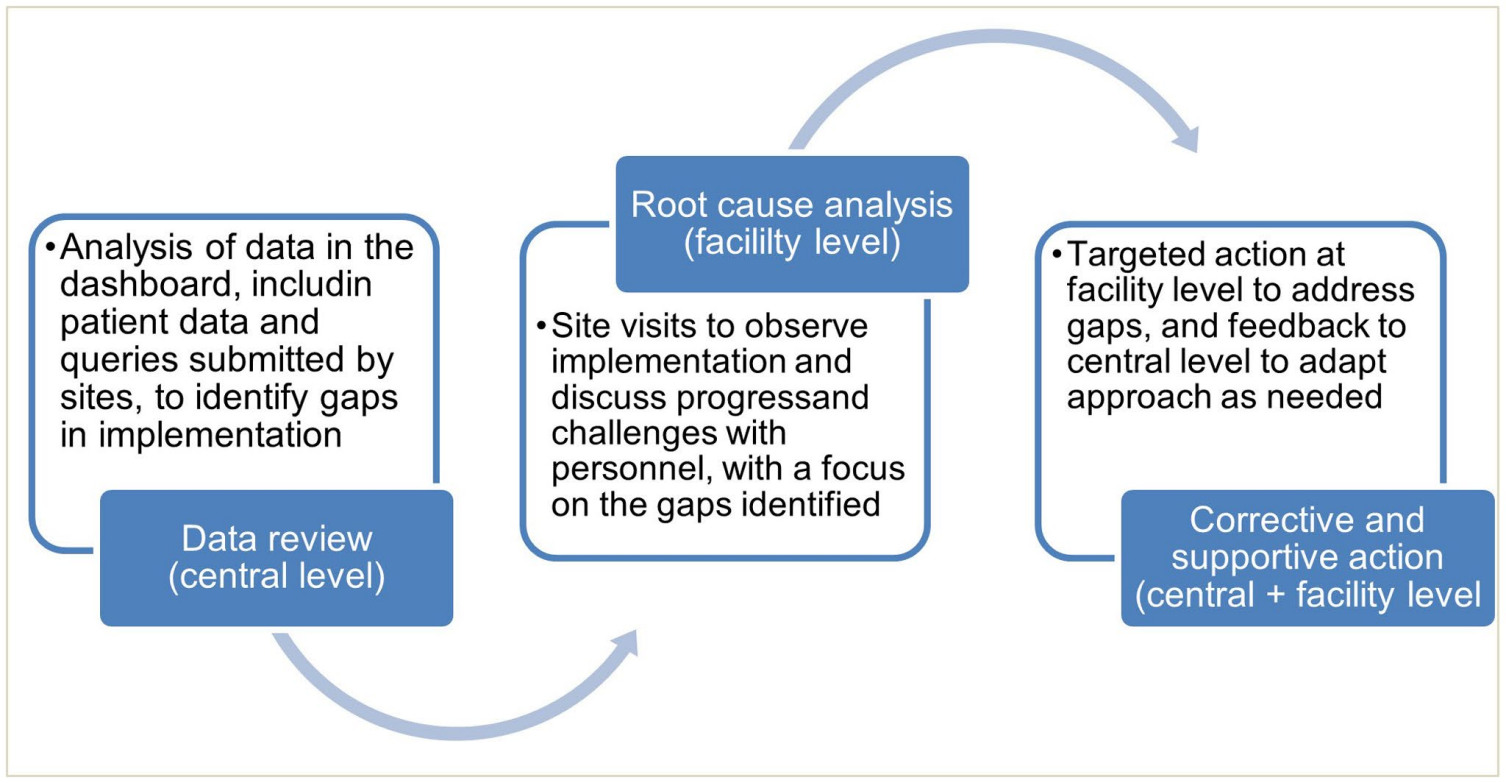
Feedback cycle informed by data from eLIF and site visits.

Monitoring was conducted through analysis of data accessed through the dashboard, together with site visits and qualitative interviews. This informed targeted actions by CPHL, where needed, for continuous quality improvement (Figure 2).

### Data analysis

Both quantitative and qualitative data were collected from the 11 health facilities. Qualitative data were collected using semi-structured questionnaires during site visits midway the study implementation period, and endpoint assessment was done using online Google forms. The quantitative dataset was downloaded from a central database in Microsoft Excel format. The dataset was cleaned and exported to STATA 14.2 for analysis.

Comparators were largely not available as most facilities do not conduct routine monitoring of the study indicators being investigated, which formed part of the rationale for introducing this digital intervention. However, if participating facilities used multiple reporting methods, comparison between different reporting methods was conducted where possible.

### Ethical approval

All study procedures were approved by the Uganda National Health Laboratory Services (UNHLS) Research Ethics Committee and the Uganda National Council for Science and Technology (UNCST), research registration number (HS1723ES), protocol amendments, or deviations during the course of implementation, were also submitted for approval.

## Results

### Uptake

#### Uptake of eLIF in testing sites

eLIF was rolled out in all 11 health facilities, ranging from health centers to referral hospitals. At the time the study was designed, COVID-19 testing was typically centralized within facilities, but as the pandemic response shifted from an emergency response to routine management, testing was increasingly disseminated across facilities. During the mid-point site visits (March 2022) it was noted that on average each health facility had two testing points, while at least two facilities were providing COVID-19 testing across all wards. Reporting practices varied both between these testing points, and across facilities. The degree of uptake of eLIF in testing sites was determined by looking at the route through which data was transmitted to RDS.

Where eLIF was not used, some healthcare workers continued to use alternative digital reporting channels such as Microsoft Excel uploads to RDS, while in some cases data were captured on COVID-19 paper registers and not transcribed to any electronic tools. The latter proportion was determined through a view of facility files during the site visits, and these data were then manually added to electronic databases. Table 1 shows the proportion of records captured through each of these methods, by facility. Overall, 64.97% of patient records were submitted via eLIF and 11.65% through alternative digital channels, while 23.38% were only recorded on paper registers and had to be manually transmitted to the national repository by study personnel during site visits (Table 2).

**Table 1:** Proportion of COVID-19 Ag RDT test records captured through different reporting methods.

Health facility	n	Count (%) of real-time data	Count (%) of .csv data	Count (%) of paper registers
Butabika RRH	880	850 (96.59)	30 (3.41)	0
Entebbe RRH	1,438	793 (55.15)	26 (1.81)	619 (43.05)
Jinja RRH	2097	338 (16.12)	1,514 (72.20)	245 (11.68)
Kawempe NRH	954	540 (56.60)	1 (0.10)	413 (43.29)
Kiruddu NRH	1,010	59 (5.84)	1 (0.10)	950 (94.06)
Mbale RRH	315	144 (45.71)	1 (0.32)	170 (53.97)
Mulago NRH	4,956	4,955 (99.98)	1 (0.02)	0
Moroto RRH	168	143 (85.12)	9 (5.36)	16 (9.52)
Soroti RRH	1897	1858 (97.94)	39 (2.06)	0
St. Mary’s Hospital, Lacor	805	144 (17.89)	159 (19.75)	502 (62.36)
Wakiso HC IV	831	150 (18.05)	7 (0.84)	674 (81.11)
Total	15,351	9,974 (64.97)	1788 (11.65)	3,589 (23.38)

(A) Real-time data were captured by health facility personnel using eLIF app within 0–3 days (min, max) with a median time of 24 h. (B) Data in .csv format were captured by health facility personnel using the Microsoft Excel spreadsheet and uploaded into RDS within 0–37 days (min, max) with a median time of 24 h. (C) Missed data were captured from COVID-19 paper registers by CPHL staff using the eLIF app during project mid-assessment as an intervention to ensure completeness of data collected at health facilities.

**Table 2:** eLIF app user activity from December 2021 to May 2022.

Summary	
Total number of users accounts issued overstudy duration	40
Total number of user-months (# of users x # of months with existing acct)	195
Proportion of all user-months that were active	50.3%
Proportion of all study-trained users still active at end of study	12.5%
Proportion of users trained at beginning (“original users”) still active at end of study	23.8%
Proportion of users deactivating during study	22.5%
Proportion of non-deactivated users still active at end of study	16.1%
Proportion of all-time users that were onboarded during study (“new users”)	0.0%
Proportion of users with inactive status at any point	92.5%
Proportion of users active for at least 70% of their user-months	22.5%

#### Utilization

The original methodology for measuring utilization assumed a largely static staff complement, with utilization to be calculated based on the proportion that regularly submitted data via eLIF. In practice, however, staff movement was more complex. Some study staff left facilities during the implementation period, while others were on occasion reassigned to other units of the facility to meet demand for other services, especially when COVID-19 testing demand decreased. This unpredictability in staff movement required an adjusted approach during the study.

To account for changes in staff workflow throughout the study, utilization was therefore determined in monthly units—calculating the number of months that all users registered in the system were active, as a proportion of the total potential months that they could have been active. Months during which users left health facilities were excluded from the denominator, with these users considered to have “deactivated” their accounts, while those who were still in the facility but not uploading data via eLIF were marked as “inactive.”

The final utilization, per user and facility, are presented in Table 2. A total of 40 user accounts were issued across the facilities during the study period. User accounts were assigned to specific COVID-19 focal persons with the responsibility of performing tests and data capture at the health facilities. Although sharing of accounts was disallowed under the study procedures, anecdotal evidence suggests that other healthcare workers may have used the focal person’s account, due to frequent and often unforeseen task-shifting to other support staff in the COVID-19 testing centers. As a result, the actual number of end-users was larger than the number of documented user accounts.

A total of 22.5% of accounts were deactivated during the study period. Overall, 50% of total potential user-months were active, with over 90% of healthcare workers being inactive at some point during the study, during which COVID-19 positivity rates declined from an initial peak during the Omicron wave of December 2021. Due to the difficulties of monitoring shifting workflows within facilities, it is possible that inactivity may have corresponded with periods during which the staff assigned to that account remained within the facility but were not conducting testing.

In the final month of the study, by which a wider drop in both positivity rates and demand for testing had taken place, only 16% of user accounts were actively submitting data.

### Effectiveness

#### Data timeliness

The main objective of eLIF was to decrease the time between testing and central results reporting. Throughout the study, the time between test being conducted and data reporting into RDS varied by collection method. Data that were captured in paper registers (23.38% of patient records) were not transcribed into any electronic reporting system, and had to be entered by CPHL staff during mid-term assessments, which were conducted 3 months after study initiation. The delay between testing date and date of entry into the register by CPHL staff varied from 0 to 130 days with a median of 24 h. Records entered via eLIF were reported within 0–3 days, with a median time of 24 h. In facilities where pre-existing reporting systems were uploaded to RDS using Microsoft Excel, a median reporting time of 24 h was also reported with a range of 0 to 37 days (min, max). In the post-study questionnaire with representatives of all facilities involved, 75% of them agreed that eLIF decreased time to results reporting, with half of these reporting strong agreement. However, 19% of respondents disagreed that the digital tool decreased reporting time, while 6% were neutral.

#### Data completeness

While eLIF strengthened results reporting, some data fields were still missing in patient records, particularly reporting of symptoms. Overall, 84% of all records submitted lacked symptom data. Among patients with positive rapid tests, across all reporting methods only 23% had complete symptom data. All symptom data came from records captured on eLIF, as other forms of reporting did not include fields for this; however, even when using eLIF healthcare workers did not always complete this step.

#### EQA

According to the study protocol, all positive samples were supposed to be referred for PCR testing as part of routine EQA, while 10% of negative samples were to be randomly selected for EQA via an automated prompt built into the app. Adherence to this procedure varied widely across facilities. Overall, only 27% of positive samples were referred for EQA, with two facilities not referring any and another three referring less than 5% of positive samples. Only two facilities (Mulago and Butabika) referred over half their positive samples for EQA.

Based on total volumes, the study met the target of testing 10% of negative samples. However, two facilities (Mulago and Kawempe) accounted for a disproportionate share of EQA volumes: contributing 42% of all negative samples, but 85% of the corresponding EQA tests on negative samples. The majority of facilities referred less than 50% of their negative tests for EQA, with site visits confirming that these referrals were not randomly selected, indicating that the eLIF selection algorithm for EQA was not effective in this study (Table 3).

**Table 3:** Eligible and referred volumes for EQA.

Health facility	Eligible Ag-RDT positive	Total Ag RDT-negative	Eligible Ag-RDT negative (10% of total)	Positive PCR tested	Negative PCR tested	% Positive PCR tested	% Negative PCR tested
Wakiso HC IV	283	548	55	0	31	0%	57%
Soroti RRH	586	1,311	131	227	35	39%	27%
Mulago NRH	725	4,232	423	392	753	54%	178%
Jinja RRH	293	1804	180	90	147	31%	81%
Entebbe RRH	424	1,013	101	15	33	4%	33%
Butabika RRH	144	736	74	83	34	58%	46%
Kiruddu NRH	232	778	78	2	28	1%	36%
Kawempe NRH	69	885	89	12	135	17%	153%
St. Mary’s Hospital, Lacor	279	526	53	11	16	4%	30%
Mbale RRH	68	247	25	0	6	0%	24%
Moroto RRH	21	147	15	4	2	19%	14%
Total	3,124	12,227	1,223	836	1,220	27%	100%

Ag-RDT, antigen rapid diagnostic test; EQA, external quality assurance; HC IV, health center 4; NRH, National Referral Hospital; PCR, polymerase chain reaction; RRH, Regional Referral Hospital. EQA tests performed from eligible and referred samples across 11 health facilities.

Overall, 836 out of 3,124 (27%) eligible Ag-RDT positive samples were referred for EQA testing. All eligible Ag-RDT negative samples were referred for EQA testing. Referral for positive EQA Ag-RDTs was low with no samples sent from Wakiso Health Centre IV and Mbale Regional Referral Hospital.

#### EQA concordance

The concordance of PCR and Ag-RDT results is shown in Table 4. A statistically significant difference was found between true positive and false negative results (value of *p* = <0.0001) as well as true negative and false positive results between health facilities. Samples with PCR cycle threshold values less than 29.99 were also significantly more likely to have false negative results. The discordance of test results was highest at day 3 since onset of symptoms.

**Table 4:** EQA concordance between PCR and Ag-RDT testing.

	True positive (TP)	False negative (FN)	*p*-value	True negative (TN)	False positive (FP)	*p*-value
Gender						
Female	382	76		489	48	
Male	318	60	0.7787	628	55	0.5806
Age (years)						
0–12	61	8		154	21	
13–18	56	7		133	6	
19–25	105	16		131	15	
26–35	216	47		234	23	
36–45	124	26		179	18	
46–55	65	18		133	11	
Above 56	72	14	0.4737	151	8	0.1555
Health facility						
Butabika Hospital	82	1		29	5	
Entebbe RRH	13	2		27	6	
Jinja RRH	57	33		137	10	
Kawempe NRH	5	7		114	21	
Kiruddu RRH	1	1		26	2	
Lacor Hospital	7	4		9	7	
Moroto RRH	4	0		2	0	
Mulago NRH	318	74		720	33	
Soroti RRH	213	14		29	6	
Mbale RRH	–	–		5	1	
Wakiso H/C IV	–	–	<0.0001***	19	12	<0.0001***
Symptoms duration (days)						
0	13	5		9	0	
1	20	9		7	0	
2	31	5		10	1	
3	41	10		9	3	
5	14	8		18	4	
6–14	24	5	0.0589	4	4	0.1068
PCR cycle threshold (CT) value						
Less than 29.99	519	98		n/a	n/a	
Above 30.00	121	37	0.0259*	n/a	n/a	

^*^Means the *p*-value has statistical significance, ^***^statistical significance representing <0.0001.

#### Improvement in services

**Figure 3:**
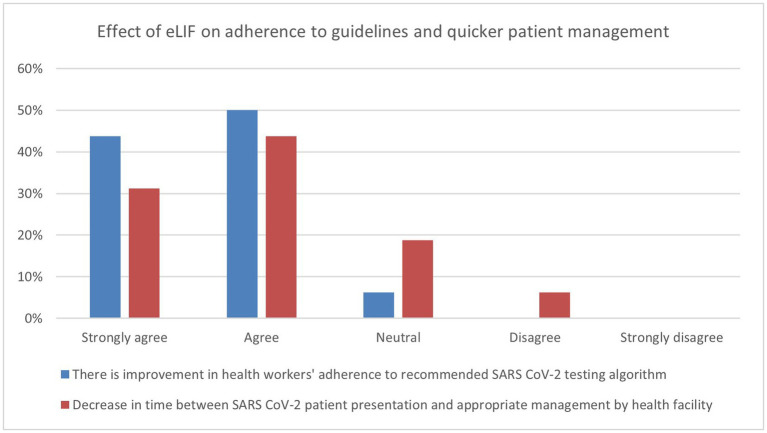
Endpoint assessment of health worker perspectives on improvement in services due to eLIF. Findings of the endpoint assessment of healthcare workers’ (*n* = 16) perspectives using eLIF in percentages. Blue = percentage responses on improvement in healthcare workers’ adherence to recommended SARS-CoV-2 testing algorithm, Red = percentage responses on decrease in time between SARS-CoV-2 patient presentation and appropriate management by health facility. SARS-CoV-2, severe acute respiratory syndrome coronavirus 2.

The majority of healthcare workers interviewed in the endpoint assessment agreed that the use of eLIF improved their adherence to diagnostic algorithms (94%). While 75% of respondents agreed that eLIF decreased the amount of time patients had to wait between presentation and appropriate management by the facility, 6% disagreed and 19% were neutral (Figure 3).

The effect of eLIF on staff workload was also assessed in the endpoint questionnaire, with healthcare workers asked whether there was a decrease in the time they spent on administrative tasks related to reporting. Three quarters of the facility representatives interviewed agreed that the efficiencies created by eLIF reduced the time spent on administrative tasks, with 31% reporting strong agreement. However, 19% disagreed with this statement and 6% were neutral.

#### Feasibility and acceptability

In the endpoint questionnaire, healthcare workers were asked whether the facility was sufficiently equipped, in terms of infrastructure and personnel, to implement eLIF. Overall, 75% of respondents agreed that their facilities had sufficient infrastructure, with 44% strongly agreeing. The perception of personnel capacity to implement eLIF was lower: only 19% strongly agreed, while 25% disagreed, that their facilities have sufficient personnel to implement eLIF.

Healthcare workers were also asked to grade their satisfaction as low, moderate or high. Half of the healthcare workers were moderately satisfied with the use of eLIF app for data collection and fitness for purpose and 31% were highly satisfied with the app. However, 19% reported low satisfaction with the eLIF app. The key challenge highlighted was instability (freezing and malfunctioning): this was flagged as the main area of improvement by half of the respondents.

## Discussion

eLIF enabled healthcare workers to capture and transmit testing data to the national reporting system more rapidly and efficiently than paper-based registers but at comparable speeds to Excel-based uploads. However, the app did not achieve the objective of creating a seamless process for collection and analysis of EQA data.

**Figure 4:**
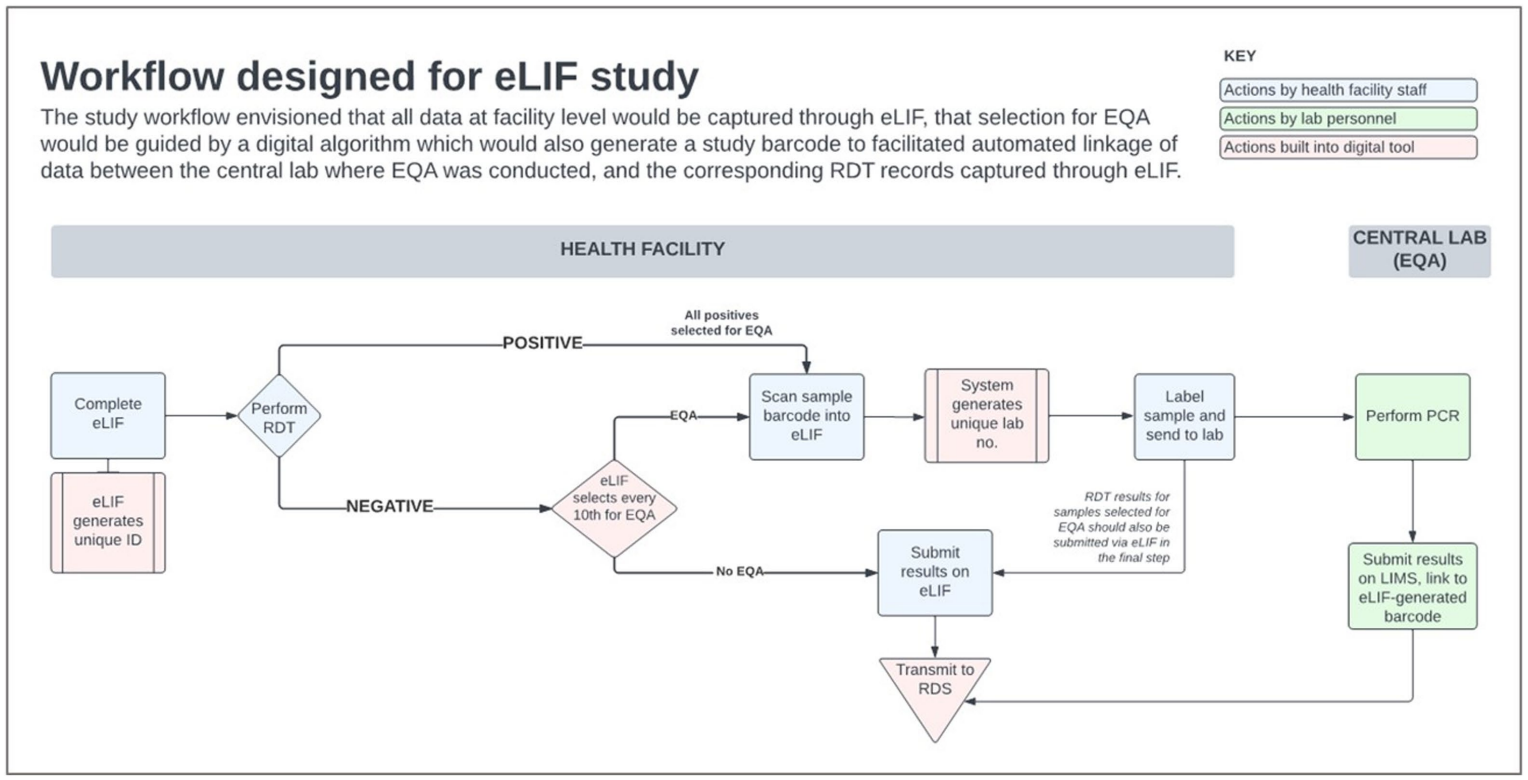
eLIF workflow. eLIF; electronic Laboratory Information Form; EQA, external quality assurance; RDS, national results dispatch system; PCR, polymerase chain reaction; Ag-RDT, antigen rapid diagnostic test.

**Figure 5:**
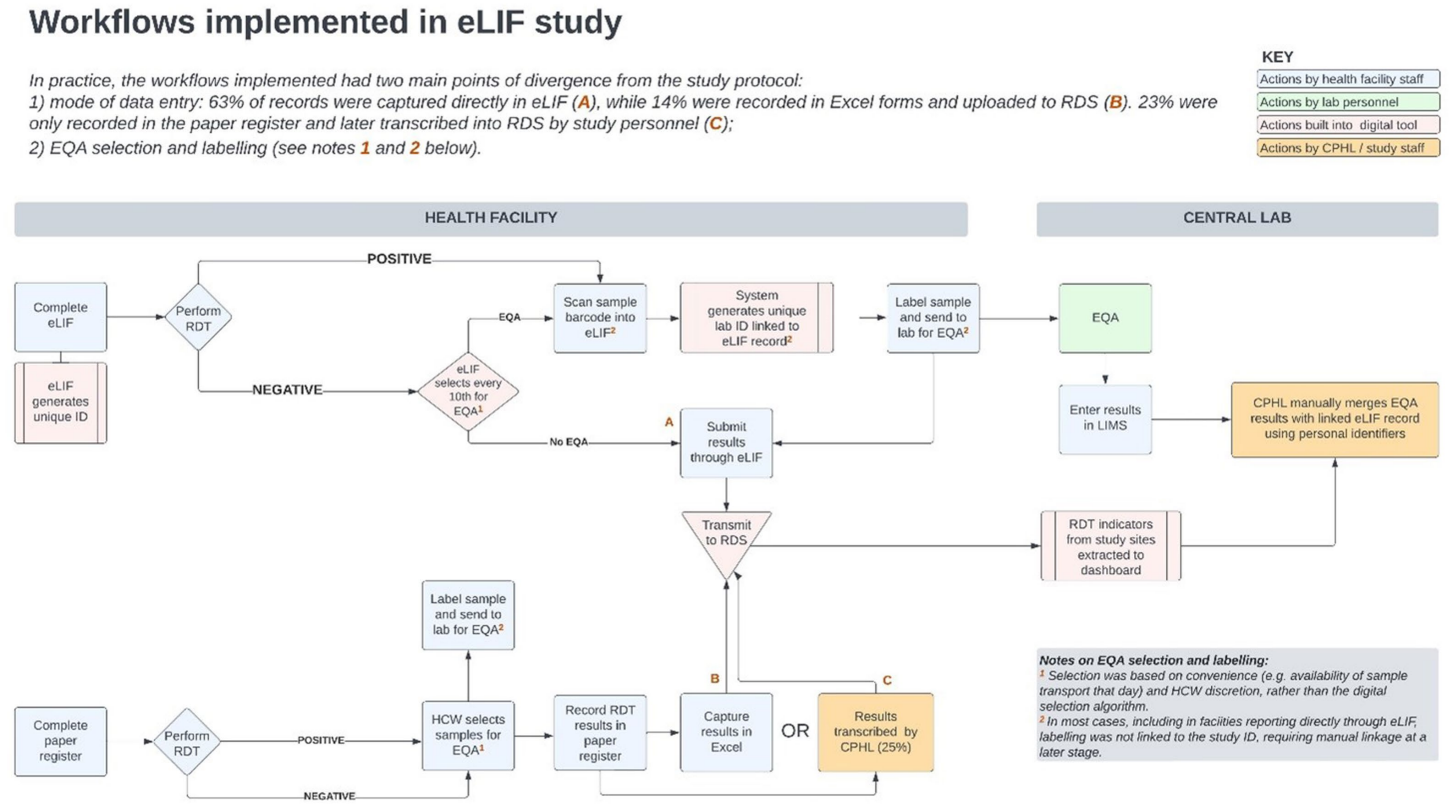
Workflows implemented in the eLIF study. CPHL, Central Public Health Laboratory; eLIF, electronic Laboratory Information Form; EQA, external quality assurance; RDS, national results dispatch system; PCR, polymerase chain reaction; RDT, antigen rapid diagnostic test.

There was extensive heterogeneity in the implementation of eLIF both across facilities, and between multiple testing points in the same facilities, highlighting the complexity of rolling out new digital tools in public health systems. Compared with the original workflow described in the methods section (Figure 4) the actual workflow implemented in the study had several points of divergence (Figure 5).

Site visits to understand the reasons behind implementation successes and challenges as observed in the data, identified several factors that determined how consistently the envisioned workflow could be implemented. These included study-related factors such as training and re-training, challenges related to the tool and availability of technical capacity to promptly resolve software challenges, and broader health systems factors such as staff turnover, personnel bandwidth for data capture, resistance to system use by service providers and availability of logistical requirements needed for adherence to the study protocol.

### Uptake

This study demonstrated that it is possible to implement real-time data capture at all levels of the health system, with participating facilities ranging from a level IV health center to a referral hospital. However, evaluation of the intervention was complicated by having multiple and frequently-shifting testing points within facilities, which changed at different stages of the study period depending on demand for testing and availability of human resources.

Using eLIF, it was not possible to detect the exact location within each facility associated with each patient record, so findings were aggregated at facility level, preventing an understanding of variations in diagnostic and reporting practices based on where testing was conducted (e.g., in the laboratory compared with the emergency room). The nature of services being provided likely influenced use of the tool—for example one facility which treats psychiatric patients, Butabika, noted that challenges around getting patients to cooperate with testing made it difficult to simultaneously do real-time data capture.

However, the most common reason given for not entering data in real-time via eLIF was workload, with traditional methods of data capture perceived to be quicker as these were systems with which personnel were familiar. Overall, around 65% of records were captured in real time via eLIF, while 12% were captured via Excel upload. Some sites reported that their reporting method varied from day to day depending on the length of the patient queue. During site visits conducted mid-way the study, it was also observed that some healthcare workers documented patient data on paper forms or Excel, and later transcribed it to eLIF. This resulted in increased workload for reporting at health facility level—and for duplicate records that were entered in both eLIF and Excel, necessitated retrospective data cleaning by CPHL. Additional implications of using different tools included lack of alignment in the data fields and formats captured, and lack of adherence to the automated process that had been built into the app for EQA sample selection and labeling.

A review of facility files found that 23% of tests that had been captured on paper registers were not transcribed into any digital tool and thus transmitted to the national repository. These “missing cases,” accounting for nearly 1 in 4 of all tests conducted, point to the importance of digital tools for providing a complete picture of diagnostic efforts and disease burden. This applied to 8 out of 10 facilities, which accounted for nearly half of all testing records (49.6%). In these sites, 47% of testing records were still captured on paper only, with eLIF and.csv accounting for 30.3 and 22.6%, respectively. In the remaining three facilities (Mulago, Soroti, and Butabika) where paper was not used, 99.1% of tests were reported through eLIF with.csv accounting for the remainder. During the facility feedback session with healthcare workers, some indicated that they experienced challenges with data entered into eLIF going “missing.” Underlying reasons for these challenges include that healthcare workers captured data offline due to limited data connectivity and users occasionally experienced limited local data storage while using personal phones. Other reasons included non-compliance with the work flow during data capture such as failing to scan the unique identifier (ID) bar codes for the EQA samples and a lack of standardized allocation of unique IDs by health facilities, whereby similar unique IDs were repeated each new day. Tablets and routers were provided to healthcare workers as a solution to these issues. In the future, it would be key to set minimum device requirements for the implementation of the eLIF application. An additional exception-handling mechanism should also be introduced in the app to prevent data loss by ensuring storage is not used up and to ensure healthcare workers can only use eLIF after offline data have been synced with the server. In addition, health facilities should also develop and generate standardized unique identification for patients. Finally, periodic communication and training on new eLIF versions should be provided to the end users. The digital system, particularly the dashboard, supported uptake by informing targeted interventions to be taken by CPHL based on analysis of performance indicators in the dashboard—including retraining of staff where needed and addressing technology challenges such as the app freezing, which discouraged personnel from using eLIF. However, some of the factors related to broader health system challenges, such as workload, were broader than the scope of the study interventions. To optimize the impact of digital tools, user-centered change management strategies are required which take into account facility and personnel workflows, as well as the operating environment.

Forty eLIF user accounts were issued over the study duration from December 2021 to May 2022, although the number of individual end-users in facilities was higher due to sharing of accounts. This was due to frequent task-shifting within facilities, with patterns that were not easy to predict or monitor. The practice of account-sharing presented a challenge for monitoring as it was difficult to measure the exact number of healthcare workers who interacted with eLIF, and where performance indicators were sub-optimal, there was a lack of accountability of data transmitted from the health facility if the specific personnel corresponding with the record could not be traced.

Only half of the total user months were active, which is notably low due to work schedule rotations and high staff turnover. This point is illustrated by the intern laboratory technologists in St. Marys’ Lacor who were trained on eLIF but all reassigned during the course of the study. Halfway into the study, at least four facilities reported that staff who had been trained were no longer conducting testing.

There was insufficient transfer of knowledge as staff shifted roles, leading to difficulties with using the tool or following study procedures. With few human resources permanently assigned to perform COVID-19 Ag-RDT testing, only 23.8% of users trained at the beginning of the study were still active at end of study, highlighting the role of periodic support supervisions and mentorship during the implementation of digital tools at health facilities.

### Effectiveness

#### Data timeliness

A key concern with decentralization of Ag-RDT testing has been the ability to access data if diagnosis is delivered outside of laboratories that have well-established reporting systems, as illustrated by the long reporting lags in other settings (11, 18). This study demonstrated the value of digital tools in enabling real-time or near real-time monitoring of decentralized testing, provided the necessary measures are put in place to facilitate implementation by healthcare workers and performance of the digital technology.

While eLIF was designed for real-time data collection and transmission, it was necessary to allow for interruptions to internet connectivity, through offline capability that allowed for data to be captured and transmitted to RDS when connectivity was restored. Due to intermittent connectivity, many records captured in eLIF were not transmitted instantaneously, and the median time between testing and reporting was therefore the same for eLIF compared with reporting via .csv (24 h).

However, records entered via CSV were delayed by up to 37 days in some cases, compared with the maximum lag between testing and reporting for eLIF, which was 3 days. By contrast, the testing records captured on paper were delayed by up to 130 days, reducing their value for COVID-19 surveillance which requires timely data.

Additionally, the majority of healthcare workers interviewed at the end of the study (75%) reported that eLIF reduced the time required to manage patients, and in facilities that had access to both reporting methods eLIF was used for the majority of tests. In sites that had multiple electronic reporting options, 65.4% of records were transmitted through eLIF while only 4.5% were transmitted through .csv and the remainder by paper, suggesting preference for use of eLIF over other reporting methods. However, in one site (Jinja), over 80% of tests were reported by entering data directly into a laboratory information system (LIS) that could readily be downloaded as a .csv file and uploaded into RDS, allowing for ease of transmission.

#### Data completeness

**Figure 6:**
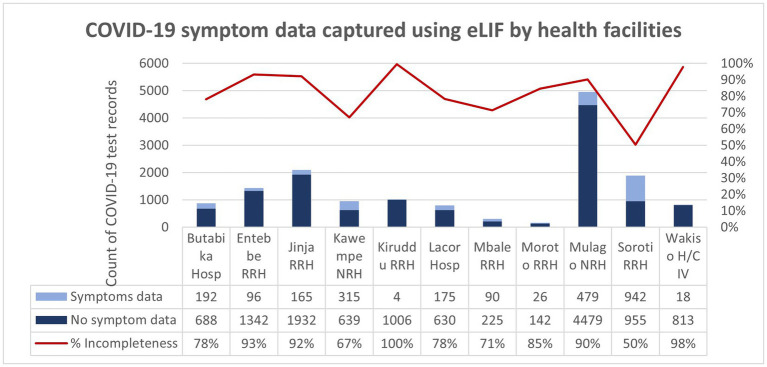
COVID-19 symptom data captured using eLIF app by health facilities. The proportion of records with incomplete symptom data (100%) was highest in Kiruddu RRH and was lowest (50%) in Soroti RRH. Overall, 84% of the COVID-19 test records transmitted using the eLIF app had incomplete COVID-19 symptom data.

The study aimed to capture symptom data that could be used to analyze correlations between clinical factors and testing outcomes to strengthen testing guidelines—for example, on more targeted selection for confirmatory PCR testing. However, symptom data were not captured for the 84% of records. This was in part due to the use of alternative reporting methods that did not have fields for data entry on symptoms, but the majority of records entered through eLIF also skipped this step. Adjustments to the tool to encourage adherence to symptom data collection could enhance data completeness and hence the insights that can be gained from this tool, for example by making it mandatory for staff to confirm that the patient does not have any symptoms in order to proceed to the next step (Figure 6).

#### EQA

The EQA selection algorithm was largely not followed, with only 1,050 referrals for PCR—51% of the expected EQA number based on the programmed procedure within eLIF. Even for these tests, selection was largely based on convenience and determined by the personnel on duty that day, rather than on the app. There were several reasons for this, summarized below.

##### Technological factors

In some testing sites, use of other modes of reporting data/results apart from eLIF meant that healthcare workers were not exposed to the selection algorithm, while intermittent malfunctioning of eLIF also contributed to lack of adherence to this procedure. It was also recommended that the selection step be made more prominent in the app—for example, by including pop-out notices for eligible EQA samples in the form of a message on screen, alert sound or change of text color.

Improper labeling also resulted in difficulty tracking samples that were referred for EQA. Unique bar codes in triplicates were allocated to each of the study facilities, which were supposed to be scanned into eLIF for every sample selected for EQA with another barcode placed on the EQA sample, for ease of linkage. Inconsistent implementation of this procedure, or facilities running out of barcodes, resulted in some EQA results not being traceable to the corresponding patient records captured through eLIF. To resolve this, manual linkage using patient names was used.

While this enabled the study team to retrieve the missing data, it required significant time compared with the automated process that had been envisioned through use of linked barcodes. Improved use of barcodes is highly recommended, as it reduces transcription errors, and enables easier linkage of records through electronic tracking systems. The intervals should be readable, water resistant, and distribution to health facilities should be monitored alongside testing demand to ensure uninterrupted supply.

##### HR-related factors

High staff turnover, and lack of sufficient training for new and remaining staff on EQA sample selection affected adherence to study procedures across all areas. High workload, due to limited number of staff available to manage large volumes of patients, also resulted in healthcare workers skipping this step. High volumes of samples selected for EQA can discourage compliance with selection procedures, as this is perceived as added work that may cause delays in the routine Ag-RDT testing. To prevent overloading healthcare workers, it may be necessary to adjust the selection algorithm to account for increased volumes or input daily maximum number of eligible EQA samples for each facility.

##### Logistics factors

Storage space and infrastructure in the facility, as well as the sample transportation schedule and availability, affected whether or not staff retained remnant samples for EQA. Availability of freezers to support cold chain temporary storage, in line with the required temperature and retention time to preserve the integrity of the remnant test, is necessary to ensure adherence to EQA algorithms for decentralized testing. There is also a need for enhanced training and supportive supervision on remnant sample management and cold chain for personnel at all testing points.

#### Improvement in services

The variations in health worker practices and health facility context were reflected in the endline feedback on how eLIF affected patient services. The strongest effect was on adherence to diagnostic algorithms, with all but one facility respondent reporting an improvement in this area. This could be due to a combination of specific guidance built into the app, and targeted supervision by CPHL based on performance gaps identified through the dashboard. It was not possible to validate healthcare worker perceptions against baseline measurements of adherence, as previous reporting methods did not allow for monitoring of this, illustrating the value of digital tools that are tailored to the insights required to inform continuous quality improvement.

Feedback on the effect of eLIF on time required for patient management and reporting was less unanimous. Overall, 75% of respondents agreed that eLIF decreased total time required for patient management, as well as time spent on administrative tasks related to reporting. However, one in five healthcare workers disagreed with the latter, experiencing increased time spent on reporting. The variation in reporting methods, particularly double data entry in some cases, may explain the increased workload experienced by some staff due to eLIF. Other contributors to increased workload could include the EQA process as explained above, training and retraining, and transitioning to a new technology that experienced some glitches during implementation.

#### Feasibility and acceptability

While no healthcare workers disagreed that the facility had sufficient infrastructure to implement eLIF (although 25% registered a neutral response), 19% responded that their facilities did not have sufficient personnel to implement the app, which may be related to the workload challenges outlined previously. Only 31% of healthcare workers reported a high degree of satisfaction with the app, while 19% reported low satisfaction and the rest, moderate satisfaction.

The main challenge reported by facility respondents was sporadic freezing of the eLIF. The technical team identified that this was due to large volumes of data being entered without assigned results. In response to this, users were advised to assign results to batches of at most 20 entries. Poor internet connectivity issues at some sites such as St. Mary’s Lacor and Moroto Regional Referral Hospital were also reported, and affected ability to transmit data with the frequency required.

While most facility respondents (88%) agreed that CPHL provided technical and troubleshooting support, this study highlighted that dedicated support at both central and facility level is important when introducing a new digital system, as existing personnel are required to support multiple tools at different levels, which would limit their capacity to provide timely support during the teething phase.

This study also highlighted the challenges of rolling out a new digital tool as part of an emergency response, which requires a more rapid development and deployment phase than would typically be expected. To optimize the design and implementation of digital interventions, a sufficient time frame is necessary to go through multiple steps of product development, including outlining detailed requirements and process evaluation for the system; conducting software development and configuration; performing robust multi-site testing and software verification processes; and finally rolling it out to staff with a go-live plan and dedicated support. Multiple factors during the pandemic made it difficult to follow this process, including increased urgency of the need for new tools, limited availability of personnel due to demands of the COVID-19 response, and mobility restrictions.

As the app was accessed via Play Store and thus subject to Google policies, this also created some additional challenges. At the start of the COVID-19 pandemic, there was an increase in demand for the development and use of Android/mobile-based applications for health services. Google developed new policies with a purpose to validate the information transmitted through the mobile applications, to mitigate the risk of false information and protect users of the applications. New policies are reviewed and shared by Google periodically, and each mobile application is obliged to adjust its settings to align with the revised policies.

The eLIF app was affected by these policy changes as it is a data capture tool for COVID-19. One of Google’s revised policies included restrictions on access to camera settings by the user, implemented by Google to ensure privacy and confidentiality. However, the eLIF app required a procedural step to scan barcodes for sample and data identification, which led to the app being rejected, and inaccessible to users via the Play Store in January 2022. During this time, the CPHL team provided app updates to facilities via an Android Package Kit and support to install these updates on users’ phones. Within a month, Google approved the use of camera settings after CPHL provided justification of the necessity of bar code scanning.

#### Implementation cost and timeline for eLIF development

While the study design did not include a detailed cost analysis, key drivers are described below to illustrate the economic considerations that would go into setting up such a system. The main cost drivers for eLIF implementation were software development (as a software developer was hired to develop, test and continually update features), hardware (internet routers and Android tablets were distributed to healthcare workers to support data capture), maintenance costs for the data center (which hosts the RDS, stores all data and avails it to users), training and mentorship supervision, and data bundles. It is difficult to compute overall work done on eLIF but tool implementation and troubleshooting took a significant amount of time. There were several eLIF implementation steps including initial supervision visits to health facilities to improve adherence to the study protocol, streamline workflow and improve real-time data capture. Thereafter, four separate virtual calls were conducted to provide feedback from monitoring visits. eLIF then underwent several modifications along with the dashboard based on Ministry of Health and Foundation for Innovative New Diagnostics (FIND) reviews. After which, a virtual stakeholder sensitization meeting was held on eLIF deployment over 1 day. An end user manual was also developed and deployed and health workers were issued with end usernames and passwords to restrict access. The dashboard was further modified to enable viewing of eLIF inputs. A customer feedback desk was established to receive end user complaints/feedback and troubleshoot any issues pertaining to eLIF. Finally, healthcare workers were trained centrally in 1 day and onsite within 5 weeks across all study facilities (between 21 October and 2 December 2021) before eLIF roll out.

During implementation, several gaps including incorrect EQA selection process and incomplete logistics data were identified through a review of study data in the dashboard, and subsequent engagement through onsite mentorship supervision with health facilities focal persons. This necessitated a mid-assessment intervention to ensure completeness of data collected at the health facilities, which entailed site visits and healthcare worker interviews using pre-structured questionnaire over a period of 4 days. Additional site visits over 4 days were conducted by Foundation for Innovative New Diagnostics (FIND) to assess compliance with study protocols and plans the assessments utilized a questionnaire focused on understanding current facility practices around COVID-19 testing and reporting. A refresher training was also provided for the main gaps identified in the first few months. Overall, this study took 9 months longer than expected due to implementation and technology issues as well as staff down time due to the pandemic.

#### Key findings

The challenges of deploying a new digital tool during a pandemic response point to the need for consistent investments in countries’ digital health architecture, as a critical component of pandemic readiness and health systems resilience. This should include deployment of interoperable, rapidly customizable tools at the point of care.

Timely and accurate reporting of information as enabled by eLIF, has advantages for Uganda’s CPHL. The data are valuable to several relevant stakeholders and are key in determining the distribution of infection rates (especially among high-risk populations, e.g., truck drivers, local, and international travelers) and transmission patterns. The data from eLIF are also important in informing COVID-19 vaccination campaigns and useful for surveillance of infection rates in schools.

Although the eLIF app was initially developed for COVID-19 data capture, it has since been adopted for use in other disease such as human African trypanosomiasis, waste-water based surveillance for COVID-19 surveillance of water bodies and at the mobile Ebola testing laboratory in Uganda. Moving forward, the eLIF app should be optimized to capture real-time and quality data for outbreak and notifiable diseases in Uganda, regionally and globally. The learnings from this intervention can be used to guide future applications of eLIF, including modifications to bridge the gaps noted in this initial roll-out. Firstly, there is a need to reduce the number of mandatory data fields to minimize the time taken to complete application workflow. As eLIF was initially derived from the paper-based Laboratory Investigation Form, it originally included data fields such as recent travel, which were relevant at the start of the pandemic but unnecessary later on. This highlights the importance of continually adapting digital data solutions to evolving realities. At a systems level, this requires mechanisms for regular alignment of data needs between policy and implementation levels, including validation exercises upon tool customization with sufficient input from health personnel at all levels of the facility to ensure the digital solution is sufficiently tailored to current realities.

Secondly, it is important to implement a bidirectional feedback function within the eLIF app that can support troubleshooting when malfunctions occur. Furthermore, it is important to ensure that there is interoperability between eLIF and the existing LIS, so that the app can be implemented smoothly. Future implementations of eLIF could also investigate the value of capturing GPS coordinates to map geographical data of where the app is used to allow insights into where testing is happening, including visualization of disease data by location and time, hotspots and disease patterns. This functionality could be a powerful real-time data source for public health responses. Development of an iOS version of the eLIF app would also increase access for international users who may use iOS devices.

In conclusion, despite some challenges with the roll-out and implementation of eLIF, the tool added value to CPHL’s efforts to monitor the decentralization of Ag-RDT testing in Uganda—most notably by improving timeliness of data in facilities that adopted eLIF which previously used paper-based reporting and providing granular visibility into implementation of diagnosis in facilities. This visibility enabled CPHL to identify and address challenges through targeted interventions. eLIF also enabled efficiency gains, particularly around staff time spent on reporting. Improvements to the tool to address the challenges experienced would enhance implementation of this tool, particularly in terms of supporting greater adherence to guidelines, preventing freezing when dealing with large volumes of data, and ongoing monitoring and calibration in response to evolving facility workflows. EQA procedures should also be designed to account for logistical and operational constraints in facilities which may prevent personnel from adhering to guidelines. More dedicated technological support is essential in the early stages of deploying an app for speedy resolution of challenges, to avoid discouraging staff from continued use of the technology.

## Data availability statement

The original contributions presented in the study are included in the article/supplementary material, further inquiries can be directed to the corresponding author.

## Ethics statement

The studies involving human participants were reviewed and approved by the Uganda National Health Laboratory Systems Research Ethics Committee and the Uganda National Council for Science and Technology (UNCST). Written informed consent from the patients/participants was not required to participate in this study in accordance with the national legislation and the institutional requirements.

## Author contributions

HN, IS, PA, RK, and OA participated in conception and design of the research work. HN and IS participated in implementation and overall study management. PN participated in the conception and review of digital health tools. JW participated in development of the eLIF mobile application. NL participated in the development of the COVID-19 dashboard. IS and OA provided technical guidance in the development of the digital health tools. HN, IS, PA, and OA participated in study monitoring and interim analysis. HN participated in quantitative data analysis. PA and KS participated in qualitative data analysis. PA wrote the manuscript. All authors contributed to the article and approved the submitted version.

## Funding

This study was funded by the Foundation for Innovative New Diagnostics (FIND), the global alliance for diagnostics.

## Conflict of interest

The authors declare that the research was conducted in the absence of any commercial or financial relationships that could be construed as a potential conflict of interest.

## Publisher’s note

All claims expressed in this article are solely those of the authors and do not necessarily represent those of their affiliated organizations, or those of the publisher, the editors and the reviewers. Any product that may be evaluated in this article, or claim that may be made by its manufacturer, is not guaranteed or endorsed by the publisher.

## Footnotes

^1^ http://covid19.cphluganda.org/
